# Codon Optimization of Insect Odorant Receptor Genes May Increase Their Stable Expression for Functional Characterization in HEK293 Cells

**DOI:** 10.3389/fncel.2021.744401

**Published:** 2021-09-06

**Authors:** Rebecca E. Roberts, Jothi Kumar Yuvaraj, Martin N. Andersson

**Affiliations:** Department of Biology, Lund University, Lund, Sweden

**Keywords:** bark beetle, codon optimization, de-orphanization, odorant receptor, HEK cell, *Ips typographus* (L.), olfaction, *Dendroctonus ponderosae*

## Abstract

Insect odorant receptor (OR) genes are routinely expressed in Human Embryonic Kidney (HEK) 293 cells for functional characterization (“de-orphanization”) using transient or stable expression. However, progress in this research field has been hampered because some insect ORs are not functional in this system, which may be due to insufficient protein levels. We investigated whether codon optimization of insect OR sequences for expression in human cells could facilitate their functional characterization in HEK293 cells with stable and inducible expression. We tested the olfactory receptor co-receptor (Orco) proteins from the bark beetles *Ips typographus* (“Ityp”) and *Dendroctonus ponderosae* (“Dpon”), and six ItypORs previously characterized in *Xenopus laevis* oocytes and/or HEK cells. Western blot analysis indicated that codon optimization yielded increased cellular protein levels for seven of the eight receptors. Our experimental assays demonstrated that codon optimization enabled functional characterization of two ORs (ItypOR25 and ItypOR29) which are unresponsive when expressed from wildtype (non-codon optimized) genes. Similar to previous *Xenopus* oocyte recordings, ItypOR25 responded primarily to the host/conifer monoterpene (+)-3-carene. ItypOR29 responded primarily to (+)-isopinochamphone and similar ketones produced by fungal symbionts and trees. Codon optimization also resulted in significantly increased responses in ItypOR49 to its pheromone ligand (*R*)-(−)-ipsdienol, and improved responses to the Orco agonist VUAA1 in ItypOrco. However, codon optimization did not result in functional expression of DponOrco, ItypOR23, ItypOR27, and ItypOR28 despite higher protein levels as indicated by Western blots. We conclude that codon optimization may enable or improve the functional characterization of insect ORs in HEK cells, although this method is not sufficient for all ORs that are not functionally expressed from wildtype genes.

## Introduction

Insect odorant receptors (ORs) are seven-transmembrane proteins expressed in olfactory sensory neurons (OSNs) within the chemosensory sensilla on the antennae (Clyne et al., 1999; Vosshall et al., 1999; Sato et al., 2008; Wicher et al., 2008). These receptors enable insects to detect an immense number of chemical signals including pheromones, plant volatiles, and microbial odors (Dahanukar et al., 2005). In order to function in the detection of odor ligands, insect ORs must be present in the OSN membrane with the olfactory receptor co-receptor known as Orco (Krieger et al., 2003; Vosshall and Hansson, 2011). Orco is highly conserved across insect species and serves at least two critical functions: chaperoning ORs to the cell membrane and forming a heteromeric cation channel with the OR which enables OSNs to transduce ligand binding into a neuronal signal (Larsson et al., 2004; Butterwick et al., 2018). Due to their role in underpinning critical behaviors, the functional characterization or “de-orphanization” of insect ORs has become a key area of research. Several heterologous expression systems are commonly used to functionally characterize ORs, including Human Embryonic Kidney (HEK) 293 cells (Corcoran et al., 2014), *Spodoptera frugiperda* Sf9 cells (Kiely et al., 2007), *Xenopus laevis* oocytes (Wagner et al., 2000), and the *Drosophila* “empty neuron system” (Dobritsa et al., 2003). Each of these systems has its own advantages and disadvantages, however, in all systems certain insect ORs are not functionally expressed. This may be due to several factors, including low gene expression, insufficient protein translation and/or trafficking to the cell membrane, or incorrect folding. HEK293 cells are frequently described as a robust and relatively low-maintenance system capable of both transient and stable expression, and production of large amounts of recombinant proteins (Thomas and Smart, 2005). As such, HEK cells have been used to characterize many insect ORs over the last several years. However, there appears to be significant variation in the levels of OR proteins in this system as indicated by Western blot analysis, and several ORs have been unresponsive to the tested odorants (Andersson et al., 2016; Yuvaraj et al., 2017, 2021; Miazzi et al., 2019a; Hou et al., 2020).

The necessary co-expression of Orco and ORs adds a layer of complexity to the functional characterization of ORs in heterologous *in vitro* expression systems, since both proteins must be present in the cell membrane at sufficient levels and properly folded. Efforts to improve the functional expression of insect ORs and Orco in HEK cells with transient expression have been undertaken. It was shown that *D. melanogaster* ORs tagged with HCN1 and rhodopsin signal peptides at the N-terminus display greater trafficking to the membrane and increased responses in HEK cells (Miazzi et al., 2019a). The use of Ca^2+^-reduced media for culturing HEK cells has also been shown to positively affect transient expression of *Locusta migratoria* ORs (Miazzi et al., 2019b). To our knowledge, however, no studies have reported any methods to improve the functional expression of insect ORs using HEK cells with inducible and stable expression (Corcoran et al., 2014).

One approach that may increase OR and Orco levels in heterologous expression systems could be the use of codon optimized gene sequences. Certain codons are used preferentially in the translation of genes to proteins by different taxa, a phenomenon known as codon usage bias (CUB) (Behura and Severson, 2013). CUB has been extensively studied and although not yet fully understood, it is affected by mutational bias, natural selection acting on translational efficiency, and genetic drift. Within a particular genome, CUB occurs due to a variety of complex factors such as gene expression, length, GC content, recombination rates, and RNA instability (Behura and Severson, 2013). A critical effect of CUB is the speed and efficiency of translation; optimal codons are translated faster and more accurately than non-optimal codons, influencing synthesis and folding of the resulting proteins (Pechmann and Frydman, 2013). This, in turn, alters protein structure and hence function (Angov et al., 2008; Bali and Bebok, 2015).

Expression of exogenous genes in heterologous systems such as HEK cells can be significantly affected by the factors contributing to codon bias, often leading to lack of expression of the gene entirely or non-functional or truncated proteins (Gustafsson et al., 2004; Angov et al., 2008). As such, it has become increasingly common to optimize the codons of genes intended for heterologous expression to the genome of the intended host organism. This is possible via free online tools, or directly from companies through which the optimized gene can be synthesized. Despite codon optimization becoming standard procedure in other areas of research, it has not been the case for the functional characterization of insect ORs (but see Miazzi et al., 2019a).

We recently used HEK cells with stable and inducible expression to functionally characterize two ORs from the Eurasian spruce bark beetle *Ips typographus* (“Ityp”) (Coleoptera; Curculionidae; Scolytinae). This species causes widespread destruction of primarily Norway spruce (*Picea abies*) forests (Schlyter and Birgersson, 1999; Raffa et al., 2016), and react to many semiochemicals including host and non-host volatiles (Zhang and Schlyter, 2003; Erbilgin et al., 2007; Andersson et al., 2010; Binyameen et al., 2014; Unelius et al., 2014), fungal symbiont odors (Kandasamy et al., 2019, 2021), and a variety of pheromone compounds (Schlyter et al., 1987, 1989, 1992; Byers, 1993). The two characterized receptors ItypOR46 and ItypOR49 responded specifically to single enantiomers of the bark beetle pheromones ipsenol and ipsdienol, respectively (Yuvaraj et al., 2021). In that study, Western blot analyses indicated a considerable difference in the cellular protein levels of these two ORs. Proteins of ItypOR46 in HEK cells were detected as a very strong band and this OR responded strongly to its main ligand (*S*)-(−)-ipsenol. In contrast, proteins levels of ItypOR49 appeared low and the response of this OR to its main ligand (*R*)-(−)-ipsdienol was weak, suggesting a correlation between OR protein levels and OR response magnitude and sensitivity. Proteins of five additional unresponsive ItypORs were barely detected from HEK cells, and these ORs were therefore not further considered in the previous study (Yuvaraj et al., 2021).

The aim of the present study was to investigate whether codon optimization could increase the protein levels and hence facilitate functional expression of insect ORs and Orcos in HEK293 cells with stable and inducible expression. We tested the Orco proteins from *I. typographus* and the mountain pine beetle *Dendroctonus ponderosae* (“Dpon”; Scolytinae) as well as six ItypORs previously de-orphanized in HEK cells and/or *Xenopus* oocytes (Hou et al., 2021; Yuvaraj et al., 2021). Our results show that codon optimization led to increases in HEK cell protein levels for seven of the eight receptors and increased ligand-induced responses in four of them, including two ORs that responded to ligands only when expressed from codon-optimized genes. We compare the response specificities of these ORs in HEK cells with their specificities in *Xenopus* oocytes and reveal both similarities and differences.

## Materials and Methods

### Codon Optimization, Cloning, and Generation of Cell Lines

Biological material, molecular cloning, and generation of TREx/ItypOrco/ItypOR HEK cell lines with non-codon optimized (wildtype, WT) receptor genes have been described previously (Yuvaraj et al., 2021). Also, the procedure of adding flanking restriction sites (5′ *Apa*I and 3′ *Not*I), Kozak sequence (cacc) and N-terminal epitope tags (Myc for Orco, V5 for ORs) to OR genes has been described (Corcoran et al., 2014). A total of six ItypOR genes (ItypOR23, 25, 27, 28, 29, and 49; GenBank accession numbers: MW556722-MW556726 and MN987211) and two Orco genes [ItypOrco (MN987209) and DponOrco] were selected for codon optimization. WT DNA sequences of the receptor genes were submitted to the Thermo Fisher Scientific GeneArt Portal and were codon-optimized for *Homo sapiens*, excluding the restriction sites, kozak sequence, starting methionine and the epitope tag. Since biological material from the North American species *D. ponderosae* was unavailable, both WT and codon optimized genes were obtained from GeneArt, with the WT gene sequence matching the Orco gene in the *D. ponderosae* genome and antennal transcriptome (Andersson et al., 2013, 2019). Resulting sequences were ligated into the pcDNA^TM^4/TO (Orco) or pcDNA^TM^5/TO (OR) expression vectors (Thermo Fisher Scientific), transformed into ampicillin-resistant HB101 competent cells (Promega), plated on agar containing ampicillin and incubated at 37°C overnight. Colonies were then screened by PCR using vector-specific primers and positive colonies were sub-cultured in LB broth with ampicillin at 37°C overnight. High yields of plasmid DNA from overnight cultures were obtained using the PureLink^TM^ HiPure Plasmid Filter Midiprep kit (Thermo Fisher Scientific), followed by Sanger sequencing at the DNA Sequencing Facility (Dept. Biology, Lund University) using the BigDye^®^ Terminator v1.1 Cycle Sequencing Kit (Thermo Fisher Scientific) to confirm the insert sequence. Plasmids containing the correct insert were then linearized overnight at 37°C using *Fsp*I, *Pci*I, or *Bst*Z17I enzymes [New England Biolabs (NEB), Ipswich, MA, United States].

Transfection of HEK cells and cell culturing followed previously described procedures (Corcoran et al., 2014). Briefly, linearized Orco genes in the pcDNA^TM^4/TO expression vector were transfected into a stable HEK293 cell line expressing an isogenic tetracycline repressor (TREx) and cultured with blasticidin antibiotic (NEB). Successfully transfected cells were selected using zeocin antibiotic (NEB). Expression and functionality of TREx/Orco cell lines was verified via Western blot and calcium fluorescence assay using the known Orco agonist VUAA1, respectively (described below). Functional TREx/Orco cell lines were then transfected with linearized ORs in the pcDNA^TM^5/TO expression vector and cultured in the presence of hygromycin B selection antibiotic (Gold Biotech). All codon optimized ItypOR genes were transfected into cells expressing the wildtype ItypOrco to test for the effects of optimizing the OR gene alone. The resulting stable cell lines were cultured, frozen and thawed using previously described methods (Corcoran et al., 2014).

### Protein Extraction and Western Blot Analysis

Cells induced to express the exogenous Orco and OR genes, as well as non-induced cells (control) were pelleted and total proteins extracted according to previously described methods (Corcoran et al., 2014). Western blot using 25 μg of total protein from each sample was subsequently performed with rabbit anti-Myc (Orco) and rabbit anti-V5 (OR) primary antibodies (1:2000), and an anti-rabbit +IgG, HRP-linked secondary antibody (1:5000) (all Cell Signaling Technology) using previously described methods (Andersson et al., 2016).

### Functional Characterization of Receptors Encoded by Wildtype and Codon Optimized Genes

Orco and OR cell lines were screened for ligand-induced activity using a previously described plate reader-based calcium fluorescence assay (Corcoran et al., 2014; Andersson et al., 2016; Yuvaraj et al., 2017). Cells were plated on poly-D-lysine coated 96-well plates (Corning) and expression of exogenous Orco and OR genes was induced using doxycycline in half the wells on each plate, leaving the remaining (non-induced) cells as negative control. Wells were loaded with the calcium-sensitive fluorophore Fluo-4AM (Life Technologies), incubated in the dark at room temperature for 30 min and washed with assay buffer prior to the assay. The screening panel included 37 ecologically relevant compounds (Supplementary Table 1), diluted in DMSO and assay buffer (Corcoran et al., 2014), and tested at 30 μM concentration in the plate wells. On each plate, the test compounds were individually added to three wells with cells induced to express the Orco and the OR gene, and three wells with non-induced control cells. Hence, each plate (biological replicate) contained three technical (well) replicates. Responsive cell lines were tested over at least three biological replicates (i.e., *n*_total_ ≥ 9) and at least two biological replicates were run for non-responding cell lines to confirm their lack of response (*n*_total_ ≥ 6). The general Orco agonist VUAA1 (Jones et al., 2011) (50 μM) was included as a positive control for functional Orco expression (but see Andersson et al., 2016; Corcoran et al., 2018), and assay buffer with 0.5% DMSO as a negative (vehicle) control. Plates were tested on the FLUOstar Omega plate reader (BMG Labtech, Ortenberg, Germany), where ligand-based activation of cells was measured as an increase in fluorescence from background readings in induced and non-induced wells. Mean ligand-induced responses (±SEM) were calculated using GraphPad Prism 6 (GraphPad Software Inc., La Jolla, CA, United States). Compounds were regarded as active if they elicited a response above 2% increase in fluorescence and only when the response in induced cells was significantly higher than that in non-induced cells. Hence, a general linear model with “induction” as a fixed factor and “plate” as a random factor (to account for inter-plate variation) was performed (IBM SPSS statistics v.23) to determine active compounds. Similar models, but using data only from induced cells, were run to test for differences in response magnitude between WT and codon optimized versions of responding receptors, or differences in response between different ligands activating the same receptor. Active compounds displaying an increase in fluorescence of 3% or more at the 30 μM screening concentration were included in subsequent dose-response experiments. Half-maximal effective concentrations (*EC*_50_) with 95% confidence intervals (C. I.) were calculated using the non-linear curve fit regression function in GraphPad Prism (version 6). Calculations of *EC*_50_ values were only performed for cell lines and compounds with (reasonably) sigmoid dose-response curves.

## Results

### Receptor Protein Detection From HEK Cells

**FIGURE 1:**
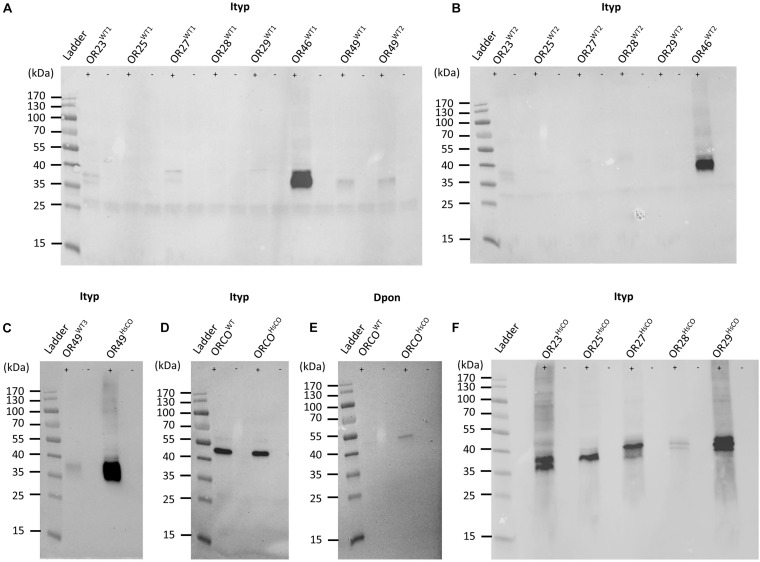
Western blots of HEK293 cells transfected with bark beetle Myc-tagged Orco genes and V5-tagged odorant receptor (OR) genes. **(A)** Detection of *Ips typographus* (Ityp) OR proteins from the first set of cell lines co-transfected with the wildtype (WT) ItypOrco gene and wildtype genes for ItypOR23, ItypOR25, ItypOR27, ItypOR28, ItypOR29, ItypOR46, and ItypOR49 (two cell lines for this gene: WT1 and WT2). Data for ItypOR46 and ItypOR49 from Yuvaraj et al. (2021) (modified image under CC BY 4.0 license; https://creativecommons.org/licenses/by/4.0/). **(B)** Detection of ItypORs from the second set of cell lines co-transfected with the wildtype ItypOrco gene and wildtype genes for ItypOR23, ItypOR25, ItypOR27, ItypOR28, ItypOR29, and ItypOR46. **(C)** Detection of ItypOR49 from cells co-transfected with the wildtype ItypOrco gene and the wildtype (third cell line: WT3) or codon optimized (HsCO) gene for ItypOR49. **(D)** Detection of Orco proteins from cells expressing the wildtype ItypOrco gene or the codon optimized ItypOrco gene, and **(E)** the wildtype *Dendroctonus ponderosae* (Dpon) Orco gene or the codon optimized DponOrco gene. **(F)** Detection of ItypORs from cells co-transfected with the wildtype ItypOrco gene and codon optimized genes for ItypOR23, ItypOR25, ItypOR27, ItypOR28, and ItypOR29. OR and Orco proteins were only detected from cells induced (+) to express the exogenous genes, and not from non-induced (−) cells, indicating proper regulation by the repressor system **(A–F)**. Note, the detection of some receptors as “double-bands” is likely due to different protein confirmations in the blotting.

We previously functionally characterized ItypOR23, 25, 27, 28, and 29 from non-codon optimized (wildtype, WT) genes using *X. laevis* oocytes, and putative key ligands were identified for all five ORs (Hou et al., 2021). Non-codon optimized genes of these five ORs were transfected into ItypOrco^WT^-expressing HEK cells for characterization also in this system. Western blot analysis of these cell lines indicated no or very low levels of OR proteins in cells (Figure 1A). To investigate whether OR protein levels in HEK cells are consistent across independent transfections of the same OR genes, the five ItypOrco^WT^/ItypOR^WT^ cell lines were produced a second time. Similar to the first set of cell lines, the ItypOR proteins were again detected as weak signals, or not at all (Figure 1B). We also generated new cell lines of ItypOR49^WT^ and ItypOR46^WT^, respectively, which were functionally characterized in HEK cells in our previous study (Yuvaraj et al., 2021). In that study, ItypOR49^WT^ (two cell lines) was detected as a faint band by Western blot, and ItypOR46^WT^ (one cell line) as an intense band (Figure 1A), correlating to the observed difference in their maximal response magnitudes. Similar Western blot band intensities were observed for the ItypOR49^WT^ and ItypOR46^WT^ expressing cells produced in the present study (Figures 1B,C). Collectively, these results suggest that OR protein levels are consistent across independent transfections and cell lines for all seven ORs included in this analysis.

The *I. typographus* Orco protein derived from the wildtype Orco gene (ItypOrco^WT^) was clearly detected from HEK cells (Figure 1D). In contrast, the Orco protein from the wildtype *D. ponderosae* Orco gene (DponOrco^WT^) was not present at a detectable level (Figure 1E).

In order to increase protein levels and facilitate functional characterization, we generated cell lines with receptor genes codon optimized for expression in human cells. The superscript HsCO (for ***H****omo* ***s****apiens* **C**odon **O**ptimized) was added after the name of these OR or Orco genes. This was performed for the above-mentioned six ItypOR genes (co-expressed in cells with ItypOrco^WT^) with no or poor protein levels in cells, as well as the ItypOrco and DponOrco genes (expressed in cells in the absence of an OR gene). The eight codon optimized receptor genes shared between 75.8 and 81% nucleotide identity with their corresponding wildtype sequences (Table 1; DNA sequences are available in Supplementary Table 2). When analyzed by Western blot, cell lines with DponOrco^HsCO^, ItypOR23^HsCO^, ItypOR25^HsCO^, ItypOR27^HsCO^, ItypOR28^HsCO^, ItypOR29^HsCO^, and ItypOR49^HsCO^ all displayed increased receptor protein levels (as indicated by band intensities) compared to the cells transfected with the corresponding wildtype receptor genes (Figures 1C,E,F). However, band intensities for DponOrco^HsCO^ and ItypOR28^HsCO^ were still comparatively faint. Results for ItypOrco^HsCO^ revealed no obvious increase compared to ItypOrco^WT^ which was already detected at high intensity (Figure 1D). Hence, our Western blot analysis suggested increased levels of seven of the eight receptor proteins when encoded by codon optimized gene sequences.

**TABLE 1:** Percent nucleotide identities of Orco and OR genes between wildtype and codon optimized sequences.

**Gene**	**ItypOrco**	**DponOrco**	**ItypOR23**	**ItypOR25**	**ItypOR27**	**ItypOR28**	**ItypOR29**	**ItypOR49**
Identity (%)	76.31	79.97	75.80	76.79	81.03	79.52	76.36	77.67

### VUAA1 Responses of Cells With Wildtype or Codon Optimized Orco Genes

**FIGURE 2:**
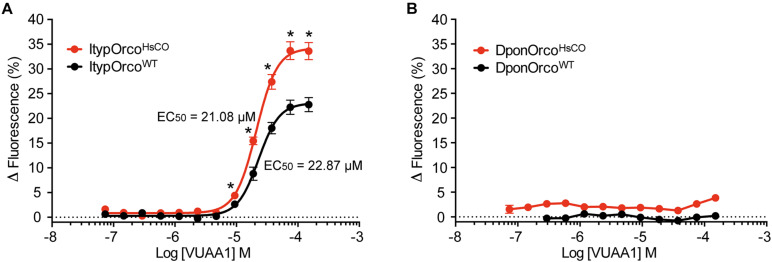
Response to VUAA1 of cells expressing bark beetle Orco genes. **(A)** Cells expressing the codon optimized *I. typographus* Orco gene (ItypOrco^HsCO^; *n* = 6 biological replicates; *n*_total_ = 18) demonstrate increased maximal response and a somewhat lower *EC*_50_ value as compared to cells expressing the wildtype Orco gene (ItypOrco^WT^; *n* = 5 biological replicates [replicates 1–3 from Yuvaraj et al. (2021)]; *n*_total_ = 15). Asterisks (*) indicate significantly higher responses in ItypOrco^HsCO^ as compared to ItypOrco^WT^ (*p* = 0.002 to <0.001). **(B)** Cells transfected with either the wildtype or codon optimized Orco gene from *D. ponderosae* (DponOrco^WT^ and DponOrco^HsCO^) failed to respond to VUAA1 (*n* = 3 biological replicates, *n*_total_ = 9), with similar Δfluorescence values in induced and non-induced cells (not shown in graph for clarity; see Supplementary Data 1). Error bars show SEM.

Cells expressing the ItypOrco^WT^ gene responded dose-dependently to VUAA1, with a maximal increase in fluorescence at approximately 23% (Figure 2A). Despite showing no obvious increase in Orco protein levels in the Western blot analysis (Figure 1D), the cell line expressing ItypOrco^HsCO^ showed a 48% higher maximal response to VUAA1, approaching 34% increase in fluorescence (Figure 2A). Responses at the five highest VUAA1 concentrations were significantly stronger in cells expressing ItypOrco^HsCO^ (all *F*_(1,26)_ ≥ 11.53; *p* = 0.002 to <0.001). Furthermore, the *EC*_50_ value was slightly lower for the codon optimized Orco (ItypOrco^WT^ *EC*_50_ = 22.87 μM, 95% C. I. = 20.30–25.76 μM; ItypOrco^HsCO^ *EC*_50_ = 21.08 μM, 95% C. I. = 19.29–23.03); however, the 95% C. I. around the *EC*_50_ was overlapping with that of the wildtype Orco, indicating this shift was not statistically significant. Cells transfected with the DponOrco^WT^ gene did not respond to VUAA1, which is in line with this protein not being detected from cells. Despite displaying an increase in protein levels (Figure 1E), cells expressing DponOrco^HsCO^ were unresponsive to VUAA1 (Figure 2B), with Δfluorescence values similar in induced and non-induced cells (Supplementary Data 1).

### Odor Responses of Cells With Wildtype or Codon Optimized OR Genes

**FIGURE 3:**
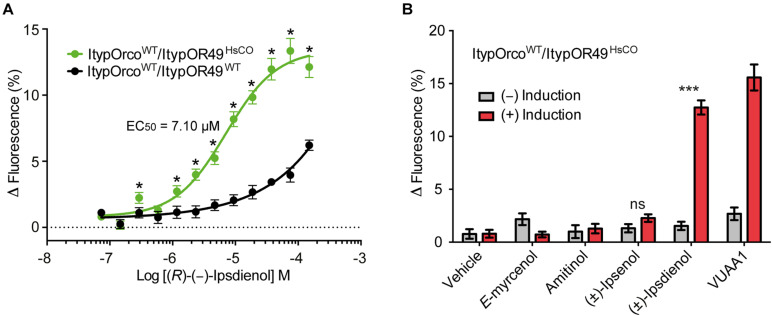
Response of cells co-expressing wildtype (WT) *I. typographus* Orco (ItypOrco^WT^) and wildtype or codon optimized (HsCO) ItypOR49. **(A)** Dose-dependent responses of ItypOR49^WT^ and ItypOR49^HsCO^ to main ligand (*R*)-(−)-ipsdienol with higher responses of cells expressing the codon optimized gene (*n* = 4 biological replicates; *n*_total_ = 12). Asterisks (*) indicate significantly higher responses in ItypOR49^HsCO^ as compared to ItypOR49^WT^ (*p* = 0.042 to <0.001). The *EC*_50_ value for ItypOR49^WT^ was not calculated due to non-sigmoid dose-response curve. **(B)** Screening results (30 μM compound concentration) from cells co-expressing ItypOrco^WT^ and ItypOR49^HsCO^ showing the same specificity as previously reported for ItypOR49^WT^ (Yuvaraj et al., 2021) (*n* = 4 biological replicates; *n*_total_ = 12). Asterisks (***) indicate significantly higher response in induced (+) versus non-induced (−) cells at *p* < 0.001. Error bars show SEM.

Cells co-expressing ItypOrco^WT^ and ItypOR49^WT^ previously responded specifically to (*R*)-(−)-ipsdienol, but responses were comparatively weak with 4.7% maximal increase in fluorescence at the highest tested concentration (Yuvaraj et al., 2021). Here, we generated a new cell line co-expressing ItypOrco^WT^ and ItypOR49^WT^. The response of this cell line was comparable to the previous data (Figure 3A). In contrast, the cell line co-expressing ItypOrco^WT^ and ItypOR49^HsCO^ displayed highly increased response magnitudes (up to fourfold, depending on concentration) to (*R*)-(−)-ipsdienol, with the strongest responses reaching above 13% increase in fluorescence. Also, our results suggest increased sensitivity of cells expressing ItypOR49^HsCO^ with significantly stronger responses to (*R*)-(−)-ipsdienol at most of the concentrations tested (all *F*_(1,19)_ ≥ 4.75; *p* = 0.042 to <0.001). Furthermore, the *EC*_50_ value of cells expressing ItypOR49^HsCO^ (*EC*_50_ = 7.10 μM, 95% C. I. = 5.56–9.78 μM; Figure 3A) was lower than that previously calculated for ItypOR49^WT^ (*EC*_50_ = 9.47 μM, 95% C. I. = 4.92–18.24 μM) (Yuvaraj et al., 2021). However, the overlap in the 95% C. I.’s indicates that this shift was not statistically significant. An *EC*_50_ value for the ItypOR49^WT^ expressing cell line assayed in the present study could not be reliably estimated due to the shape of the dose-response curve. To investigate whether the increased responses of ItypOR49^HsCO^ would reveal responses to the secondary ligands that activate the putatively corresponding OSN class (Andersson et al., 2009), we also tested the OSN-active compounds (±)-ipsenol, amitinol, and *E*-myrcenol at the 30 μM screening concentration. No significant response to these compounds were triggered in the OR (Figure 3B), although a tendency was apparent for (±)-ipsenol (*F*_(1,14)_ = 3.91; *p* = 0.068). Hence, this cell line presented the same odor specificity of ItypOR49 as previously reported (Yuvaraj et al., 2021), with only ipsdienol eliciting a significantly higher response in induced compared to non-induced cells (*F*_(1,14)_ = 231.7; *p* < 0.001).

**FIGURE 4:**
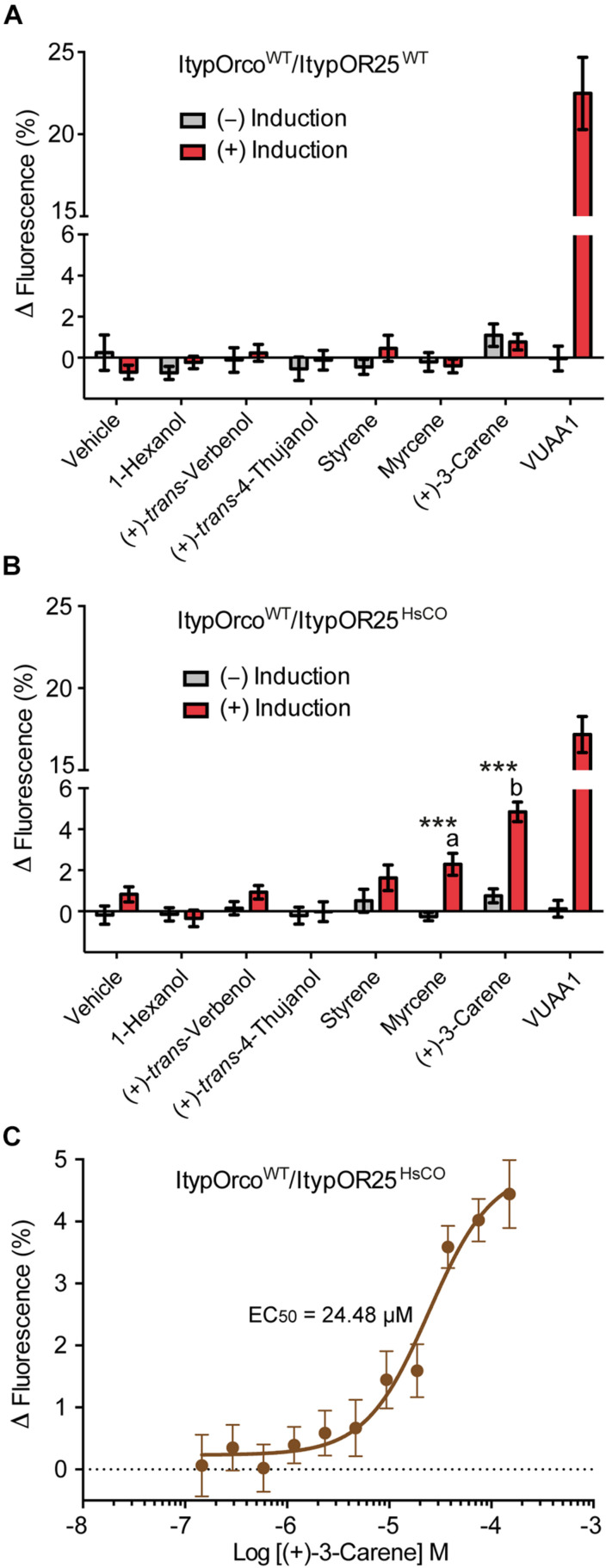
Response of cells co-expressing wildtype *I. typographus* Orco (ItypOrco^WT^) and wildtype or codon optimized (HsCO) ItypOR25 to select compounds. **(A)** ItypOR25^WT^ did not respond to any compound in the screening experiments (30 μM concentration; *n* = 2 biological replicates; *n*_total_ = 6). **(B)** ItypOR25^HsCO^ responded primarily to (+)-3-carene and secondarily to myrcene in the screening experiment (*n* = 3 biological replicates, *n*_total_ = 9). Asterisks (***) indicate significantly higher response in induced (+) versus non-induced (−) cells at *p* < 0.001, and different lowercase letters indicate significant differences between compounds at *p* < 0.001. **(C)** Dose-dependent response of ItypOR25^HsCO^ to (+)-3-carene (*n* = 3 biological replicates; *n*_total_ = 9). Error bars show SEM. Responses of ItypOR25^WT^ and ItypOR25^HsCO^ to the full odor panel are presented in Supplementary Figure 1.

ItypOR25^WT^ was recently shown to respond primarily to the conifer/host compound (+)-3-carene and secondarily to several additional compounds when tested in *Xenopus* oocytes (Hou et al., 2021). Here, HEK cells transfected with the ItypOR25^WT^ gene did not respond to any stimulus, which correlates to its low protein levels in cells (Figure 4A). In contrast, cells transfected with the ItypOR25^HsCO^ gene responded to (+)-3-carene with significantly stronger responses in induced compared to non-induced cells (*F*_(1,14)_ = 53.9; *p* < 0.001; Figure 4B), and a secondary response was elicited by myrcene (*F*_(1,14)_ = 21.4; *p* < 0.001). The response to myrcene was significantly weaker than that to the primary ligand (+)-3-carene (*F*_(1,14)_ = 19.6; *p* < 0.001). Subsequent experiments showed a dose-dependent response to (+)-3-carene (myrcene not tested due to low screening response) with an estimated *EC*_50_ value of 24.48 μM (95% C. I. = 12.46–48.13 μM) (Figure 4C).

**FIGURE 5:**
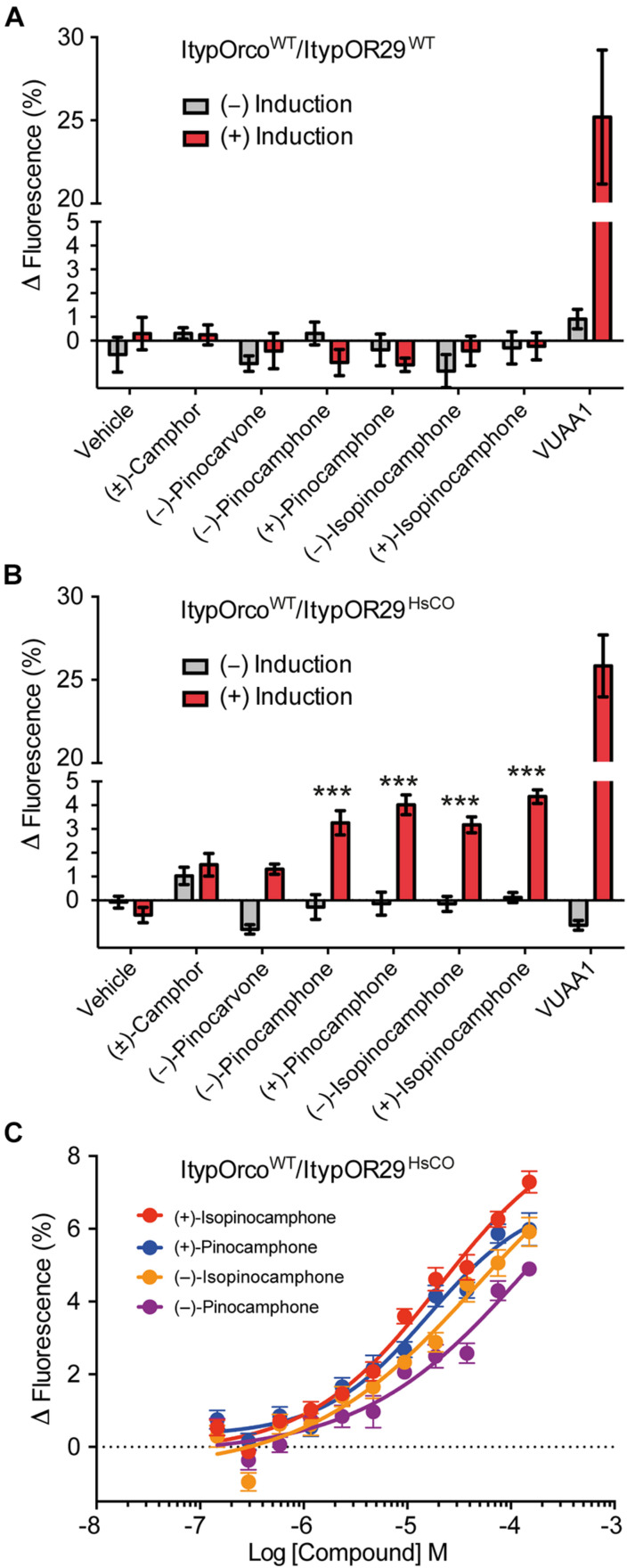
Response of cells co-expressing wildtype *I. typographus* Orco (ItypOrco^WT^) and wildtype or codon optimized (HsCO) ItypOR29 to select compounds. **(A)** ItypOR29^WT^ did not respond to any compound in the screening experiments (30 μM concentration; *n* = 2 biological replicates; *n*_total_ = 6). **(B)** ItypOR29^HsCO^ responded primarily to (+)-isopinocamphone followed by (+)-pinocamphone, (−)-pinocamphone, and (−)-isopinocamphone in the screening experiment (*n* = 3 biological replicates, *n*_total_ = 9). Asterisks (***) indicate significantly higher response in induced (+) versus non-induced (−) cells at *p* < 0.001. Responses to the four active compounds were not significantly different (*p* = 0.059). **(C)** Dose-dependent response of ItypOR29^HsCO^ to the four active ligands (*n* = 3 biological replicates; *n*_total_ = 9). Error bars show SEM. *EC*_50_ values were not calculated due to non-sigmoid dose-response curves. Responses of ItypOR29^WT^ and ItypOR29^HsCO^ to the full odor panel are presented in Supplementary Figure 2.

ItypOR29^WT^ previously responded best to (+)-isopinocamphone (produced by symbiotic fungi and the conifer host tree) when tested in *Xenopus* oocytes (Hou et al., 2021). Secondary, yet strong, responses were triggered by the structurally similar ketones (−)-isopinocamphone, (+)-pinocamphone, (−)-pinocamphone, (−)-pinocarvone, and (±)-camphor. The HEK cell line transfected with ItypOR29^WT^ did not respond to any compound (Figure 5A), and this cell line indicated low OR protein levels. However, similar to the responses in oocytes, HEK cells expressing ItypOR29^HsCO^ responded primarily and dose-dependently to (+)-isopinocamphone (*F*_(1,14)_ = 185.6; *p* < 0.001), followed by (+)-pinocamphone (*F*_(1,14)_ = 138.1; *p* < 0.001), (−)-pinocamphone (*F*_(1,14)_ = 73.8; *p* < 0.001), and (−)-isopinocamphone (*F*_(1,14)_ = 47.8; *p* < 0.001). However, the differences in responses elicited by the four active compounds were marginally non-significant at the 30 μM screening concentration (*F*_(3,30)_ = 2.76; *p* = 0.059; Figures 5B,C). In contrast to oocytes, no response was elicited by (−)-pinocarvone or (±)-camphor.

Neither wildtype nor codon optimized versions of ItypOR23, ItypOR27 and ItypOR28 showed any response to their proposed key ligands (*trans*-4-thujanol, *p*-cymene, and *E*-myrcenol, respectively; from *Xenopus* oocytes) (Hou et al., 2021), or any other tested compound (Supplementary Data 1), although cells with codon optimized genes displayed increased OR protein levels in the Western blot analysis.

All cell lines co-expressing ItypOrco^WT^ and any of the ItypORs showed strong responses to VUAA1, indicating functional expression of the Orco protein (Figures 3–5; Supplementary Data 1). Raw data from all assays and cell lines are reported in Supplementary Data 1.

## Discussion

The aim of this study was to determine whether beetle Orcos and ORs would display increased protein levels and hence increased responses to known ligands when their genes are codon-optimized for expression in HEK293 cells. Of the eight genes tested, seven displayed observable increases in cellular protein levels after codon optimization as indicated by Western blot. Four receptors from codon optimized genes displayed increased responses – or showed a response when previously there was none – to their ligands in calcium-fluorescence assays, when compared to their wildtype counterparts.

The potential benefits of using codon optimized receptor genes is perhaps best evidenced in ItypOR25^HsCO^ and ItypOR29^HsCO^, for which this approach alone enabled their functional characterization in HEK cells. While the protein levels of these ORs when encoded from wildtype genes were insufficient to respond to ligands, the ORs from codon-optimized genes were robustly detected and responded to the primary ligands previously discovered (Hou et al., 2021). Additionally, ItypOR49^HsCO^ showed both increased protein levels and significantly increased responses to its key ligand (*R*)-(−)-ipsdienol (Yuvaraj et al., 2021), as compared to ItypOR49^WT^. Codon optimization also increased the performance of ItypOrco, although the non-optimized version already responds strongly to VUAA1 and is detected at similar Western blot band intensity. Hence, our results suggest that codon optimization could be a useful tool for the functional characterization of insect ORs in the stably expressing and inducible HEK293 cell system (Corcoran et al., 2014). Indeed, in other research areas codon optimization is a default method for expression of genes in heterologous systems. The scarcity of studies (Miazzi et al., 2019a) employing codon optimization for functional characterization of insect ORs is therefore surprising, particularly when several studies utilizing HEK cells have reported non-responding ORs with seemingly low protein levels (e.g., Andersson et al., 2016; Yuvaraj et al., 2017).

However, it is also evident from our results that codon optimization of insect OR or Orco genes is not always sufficient for functional expression in HEK cells, and it may have to be complemented with other optimization methods (Miazzi et al., 2019a). For example, the observed increases in protein levels of both ItypOR28^HsCO^ and DponOrco^HsCO^ were small, and apparently insufficient for functional characterization. The reason why codon optimization had minimal effects on the protein levels of these particular receptors remains unknown. More surprisingly, both ItypOR23^HsCO^ and ItypOR27^HsCO^ failed to respond to their reported key ligands despite indicating large increases in protein levels. This demonstrates that other factors than cellular protein levels are essential, such as proper trafficking and assembly of the receptor complex in the cell membrane (Miazzi et al., 2019a) or the folding of the protein in the host cell. It is also apparent that the relationship between cellular protein levels and response to ligands is not linear. For example, the codon optimized ItypOrco did not display an obvious increase in protein levels, yet it showed significantly higher responses to VUAA1 as compared to the wildtype ItypOrco. While the absence of observable differences in protein levels in this particular case could be due to methodological limitations (i.e., both ItypOrco^WT^ and ItypOrco^HsCO^ were detected at essentially saturated band intensities), previous work on other ORs and Orcos have shown robust ligand-induced responses also from receptors with comparatively weak Western blot band intensities (Corcoran et al., 2018). Together, the results of the present and previous studies indicate that multiple processes contribute to these variable outcomes.

It also remains unknown why several insect ORs previously characterized in HEK cells show high protein levels and strong ligand-induced responses when expressed from wildtype genes, and hence do not need to be codon optimized. For instance, ItypOR46^WT^ responds robustly and is detected at similar Western blot band intensities as several of the codon optimized receptors analyzed here (Yuvaraj et al., 2021). One may assume that the wildtype sequence of this receptor gene would be more similar to its codon optimized counterpart as compared to those receptor genes that need to be optimized for functional expression. However, this is not the case since the DNA sequence identity between ItypOR46^WT^ and ItypOR46^HsCO^ is 77%, which is comparable to the identities of the eight receptors that were codon optimized and tested (Table 1). This observation may suggest that removal of certain detrimental nucleotide motifs in the codon optimization process may be more important than the absolute or relative number of altered nucleotides.

The ItypORs investigated in this study were previously characterized in *Xenopus* oocytes (except for ItypOR49 which was not functional in this system) with response specificities generally consistent with the putatively corresponding antennal OSN classes (Andersson et al., 2009; Kandasamy et al., 2019, 2021; Schiebe et al., 2019; Hou et al., 2021). Here, codon optimization enabled the characterization of ItypOR25 and ItypOR29 also in HEK cells, and the primary ligands [(+)-3-carene and (+)-isopinocamphone, respectively] are the same in both expression systems. For ItypOR29, the three most active secondary ligands (similar ketones) are also the same in both systems, however, ItypOR29 shows a broader tuning in oocytes with additional ligands eliciting minor responses (Hou et al., 2021). The system-dependent response is, however, larger for ItypOR25. The specificity of this OR in HEK cells is rather consistent with the high specificity of the putatively corresponding OSN class although myrcene is inactive on the OSNs (Andersson et al., 2009). In contrast, this receptor responded to several additional ligands in oocytes (Hou et al., 2021). System-dependent responses have now been reported for several ORs from both bark beetles (Yuvaraj et al., 2021) and moths (Hou et al., 2020). Whereas the primary ligands for such ORs are generally the same in both oocytes and HEK cells, the OR tuning breadth and rank order between the secondary ligands differ quite frequently, and there is still no consensus of in which system the OR specificities best match the *in vivo* OSN specificities. The reasons for the OR response variation between heterologous expression systems remain unknown but could potentially be due to differences in cell membrane composition affecting protein structure and access of ligands to the binding site.

This study on bark beetle olfactory receptors addressed the effects of codon optimizing the Orco gene or optimizing OR genes and co-expressing these with a non-optimized Orco gene. Overall, our results show that codon optimization generally increased HEK cell protein levels of the investigated insect receptors, and that functional expression was enabled or improved in half of the cases. Despite the variable outcomes, we suggest that codon optimization should become more widely used in insect OR de-orphanization employing non-insect *in vitro* systems to increase success rate and reduce “false negatives.” Several avenues remain to be explored, such as testing the effect of codon optimization on ORs from additional insect orders, and the combined effect of simultaneously increasing receptor protein levels and trafficking.

## Data Availability Statement

The original contributions presented in the study are included in the article/Supplementary Material, further inquiries can be directed to the corresponding author.

## Author Contributions

RR and MA conceived, conceptualized, designed the study, drafted the manuscript, and produced the final figures. RR and JY performed molecular work, cell culturing, Western blots, and functional assays. MA performed statistical analysis. All authors contributed to manuscript editing, read, and approved the submitted version.

## Conflict of Interest

The authors declare that the research was conducted in the absence of any commercial or financial relationships that could be construed as a potential conflict of interest.

## Publisher’s Note

All claims expressed in this article are solely those of the authors and do not necessarily represent those of their affiliated organizations, or those of the publisher, the editors and the reviewers. Any product that may be evaluated in this article, or claim that may be made by its manufacturer, is not guaranteed or endorsed by the publisher.
