# Supplementary material for: Codon Optimization of Insect Odorant Receptor Genes May Increase Their Stable Expression for Functional Characterization in HEK293 Cells
Source: Front Cell Neurosci. 2021 Sep 6;15:744401. doi: 10.3389/fncel.2021.744401 (PMC8450354; doi:10.3389/fncel.2021.744401)
Supplement: Supplementary file 1 [file Table_1.DOCX]

**Supplementary Table 1.** Compounds used for characterization of odorant receptors (ORs) and Orcos, including their purities, source information, and examples of main biological origins.

| **Compound** | **Purity (%)** | **Source** | **Examples of biological origins** |
| --- | --- | --- | --- |
| Acetophenone | 99 | Acros | Beetle, fungi |
| Amitinol | 91 | R. U. | Beetle |
| (±)-Camphor | 97 | Aldrich | Host, fungi |
| (+)-3-Carene | 99 | Aldrich | Host |
| 1,8-Cineole | 99 | Aldrich | Host |
| (5*S,*7*S*)-*trans*-Conophthorin | 94 | W. F. | Non-host, fungi |
| *p*-Cymene | 99 | Acros | Host |
| 3,4-Dimethoxytoluene | 98 | Givaudan-Roure | Host, fungi |
| Estragole | 99 | Aldrich | Host, fungi |
| 4-Ethylguaiacol | 98 | Sigma-Aldrich | Fungi |
| Eugenol methyl ether | 99 | Fluka | Host, fungi |
| Geranylacetone | 99 | Fluka | Non-host, fungi |
| 1-Hexanol | 99 | Fluka | Non-host, fungi |
| (±)-Ipsdienol | 94 | Bedoukian | Beetle |
| (−)-Ipsdienol | 99 (98% ee) | A. M. | Beetle |
| (±)-Ipsenol | 95 | Synergy Semiochemicals | Beetle |
| (+)-Isopinocamphone | 99 | R. U. | Host, fungi |
| (−)-Isopinocamphone | 99 | R. U. | Host, fungi |
| Lanierone | 99 | Synergy Semiochemicals | Beetle |
| 2-Methyl-3-buten-2-ol | 99 | Acros | Beetle, fungi |
| Myrcene | 95 | Sigma-Aldrich | Host |
| *E*-myrcenol | 99 | Fytofarm | Beetle |
| (±)-3-Octanol | 97 | Sigma-Aldrich | Non-host, fungi |
| (±)-1-Octen-3-ol | 98 | Janssen Chimica | Non-host, fungi |
| 2-Phenylethanol | 99 | Sigma | Beetle, fungi |
| (+)-α-Pinene | 98 | Janssen Chimica | Host |
| (+)-Pinocamphone | 84 (16% IPC) | R. U. | Host, fungi |
| (−)-Pinocamphone | 81 (19% IPC) | R. U. | Host, fungi |
| (−)-Pinocarvone | 99 | Y. N. | Beetle, fungi |
| Styrene | 99 | Fluka | Fungi |
| γ-Terpinene | 97 | Aldrich | Host |
| (+)-*trans*-4-Thujanol | 97 | Sigma-Aldrich | Host, fungi |
| (−)-(4*S*)-*cis*-Verbenol | 95 | Borregaard | Beetle |
| (−)-*trans*-Verbenol | 97 | SciTech Ltd., Prague | Beetle |
| (+)-*trans*-Verbenol | 92 | SCM | Beetle |
| (−)-Verbenone | 99 | Fluka | Beetle, fungi |
| 4-Vinyl anisole | 97 | Aldrich | Fungi |
| VUAA1 | 98 | Sigma-Aldrich | None |

Abbreviations: ee = enantiomeric excess; IPC = isopinocamphone; R. U. = gift from Rikard Unelius (Linnaeus University, Kalmar, Sweden); W. F. = gift from Wittko Francke (University of Hamburg, Germany); A. M. = gift from Aleš Machara (Academy of Sciences of the Czech Republic, Prague); Y. N. = gift from Yoko Nakamura (Max Planck Institute for Chemical Ecology, Jena, Germany).
