# Supplementary material for: Codon Optimization of Insect Odorant Receptor Genes May Increase Their Stable Expression for Functional Characterization in HEK293 Cells
Source: Front Cell Neurosci. 2021 Sep 6;15:744401. doi: 10.3389/fncel.2021.744401 (PMC8450354; doi:10.3389/fncel.2021.744401)
Supplement: Supplementary file 4 [file Image_1.PDF]

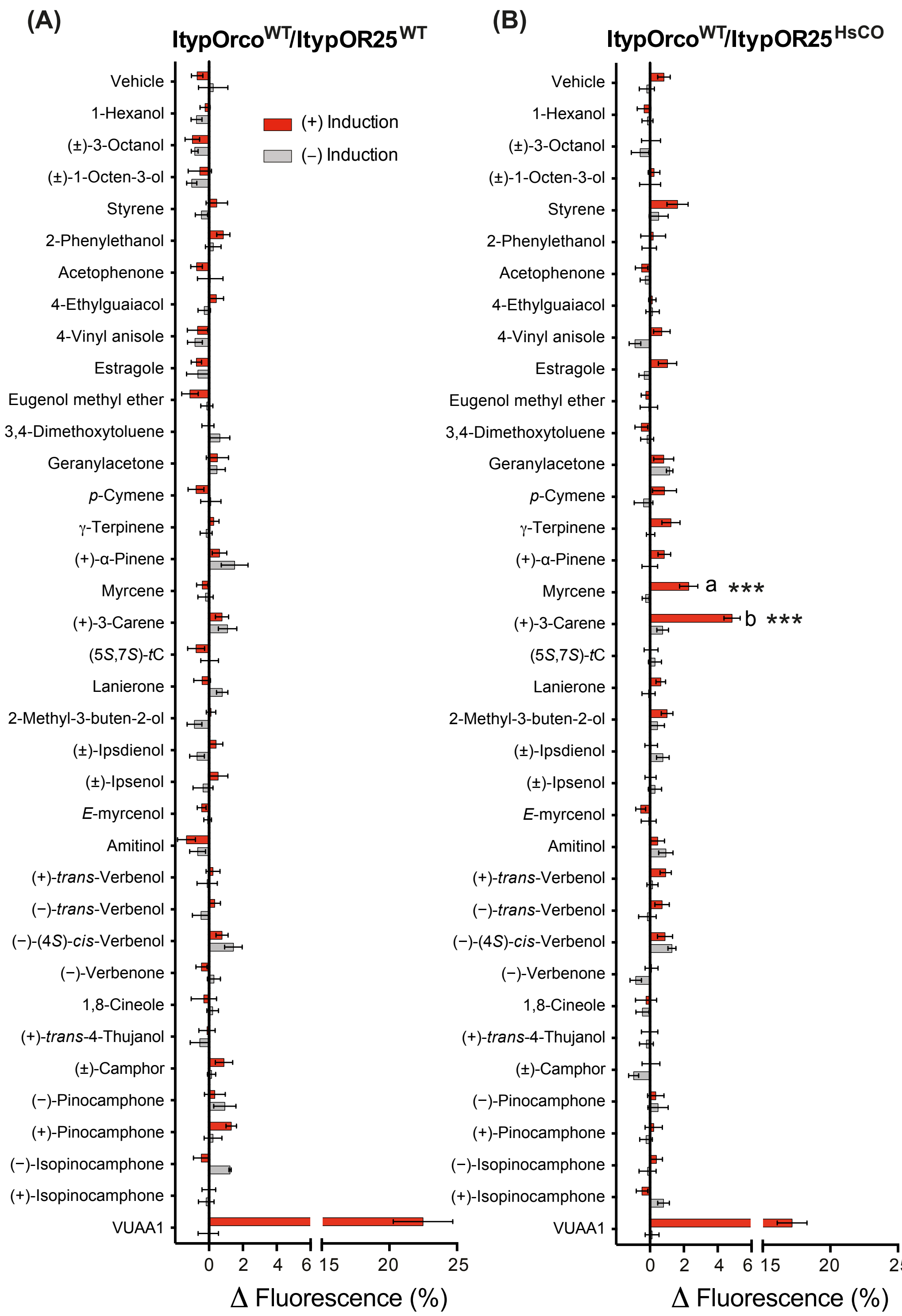

Supplementary Figure 1. Response of cells co-expressing wildtype *I. typographus* Orco (ItypOrco^{WT}) and wildtype or codon optimized (HsCO) ItypOR25 to the full test odor panel. (A) ItypOR25^{WT} did not respond to any compound in the screening experiments (30 µM concentration; n = 2 biological replicates; n_{total} = 6). **(B)** ItypOR25^{HsCO} responded primarily to (+)-3-carene and secondarily to myrcene in the screening experiment (n = 3 biological replicates; n_{total} = 9). Asterisks (***) indicate significantly higher response in induced versus non-induced cells at $p < 0.001$, and different lowercase letters indicate significant differences between compounds at $p < 0.001$. Error bars show SEM. Abbreviation: (5*S*,7*S*)-*t*C = (5*S*,7*S*)-*trans*-conophthorin.
