# Supplementary material for: Codon Optimization of Insect Odorant Receptor Genes May Increase Their Stable Expression for Functional Characterization in HEK293 Cells
Source: Front Cell Neurosci. 2021 Sep 6;15:744401. doi: 10.3389/fncel.2021.744401 (PMC8450354; doi:10.3389/fncel.2021.744401)
Supplement: Supplementary file 5 [file Image_2.pdf]

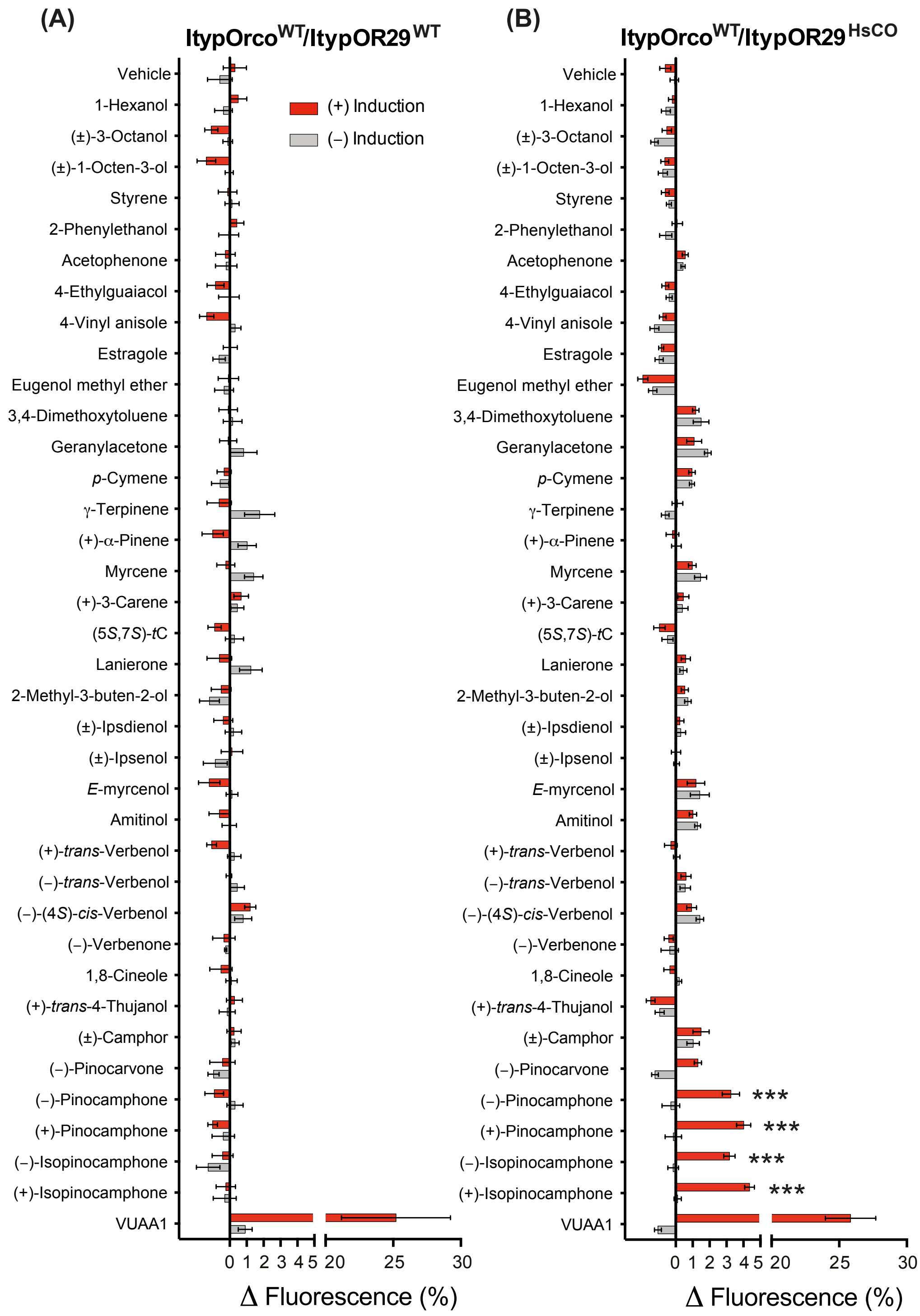


**Supplementary Figure 2. Response of cells co-expressing wildtype *I. typographus* Orco (ItypOrco$^{WT}$) and wildtype or codon optimized (HsCO) ItypOR29 to the full test odor panel. (A)** ItypOR29$^{WT}$ did not respond to any compound in the screening experiments (30 µM concentration; n = 2 biological replicates; $n_{total} = 6$). **(B)** ItypOR29$^{HsCO}$ responded primarily to (+)-isopinocamphone followed by (+)-pinocamphone, (–)-pinocamphone, and (–)-isopinocamphone in the screening experiment (n = 3 biological replicates; $n_{total} = 9$). Asterisks (***) indicate significantly higher response in induced versus non-induced cells at $p < 0.001$. Responses to the four active compounds were not significantly different ($p = 0.059$). Error bars show SEM. Abbreviation: (5*S*,7*S*)-*t*C = (5*S*,7*S*)-*trans*-conophthorin.
